# Voice-Message–Based mHealth Intervention to Reduce Postoperative Penetrative Sex in Recipients of Voluntary Medical Male Circumcision in the Western Cape, South Africa: Protocol of a Randomized Controlled Trial

**DOI:** 10.2196/resprot.5958

**Published:** 2016-07-26

**Authors:** Sarah C Thomsen, Donald Skinner, Yoesrie Toefy, Tonya Esterhuizen, Michael McCaul, Max Petzold, Vinod Diwan

**Affiliations:** ^1^ Global Health Department of Public Health Sciences Karolinska Institutet Solna Sweden; ^2^ Department of Interdisciplinary Sciences Faculty of Medicine and Health Sciences University of Stellenbosch Tygerberg South Africa; ^3^ Centre for Evidence-based Health Care Faculty of Medicine and Health Sciences Stellenbosch University Tygerberg South Africa; ^4^ Health Metrics Unit Sahlgrenska Academy Gothenburg University Gothenburg Sweden

**Keywords:** protocol, RCT, male circumcision, HIV, mHealth, VMMC

## Abstract

**Background:**

There is an increased risk of transmission of sexually transmitted infections (STIs), including HIV, in the postoperative period after receiving voluntary medical male circumcision (VMMC). In South Africa, over 4 million men are being targeted with VMMC services but the health system is not able to offer quality counseling. More innovative strategies for communicating with and altering behavior in men and their partners in the postoperative period after VMMC are needed.

**Objective:**

This paper presents a study protocol to test the effectiveness of an mHealth intervention designed to task-shift behavior change communication from health care personnel to an automated phone message system, encouraging self-care.

**Methods:**

A single-blind, randomized controlled trial will be used. A total of 1188 participants will be recruited by nurses or clinicians at clinics in the study districts that have a high turnover of VMMC clients. The population will consist of men aged 18 years and older who indicate at the precounseling session that they possess a mobile phone and consent to participating in the study. Consenting participants will be randomized into either the control or intervention arm before undergoing VMMC. The control arm will receive the standard of care (pre- and postcounseling). The intervention arm will received standard of care and will be sent 38 messages over the 6-week recovery period. Patients will be followed up after 42 days. The primary outcome is self-reported sexual intercourse during the recovery period. Secondary outcomes include nonpenetrative sexual activity, STI symptoms, and perceived risk of acquiring HIV. Analysis will be by intention-to-treat.

**Results:**

Enrollment is completed. Follow-up is ongoing. Loss to follow-up is under 10%. No interim analyses have been conducted.

**Conclusions:**

The intervention has the potential of reducing risky sexual behavior after VMMC. The platform itself can be used for many other areas of health that require task shifting to patients for better efficiency and access.

**Trial Registration:**

Pan-African Clinical Trial Registry: PACTR201506001182385

## Introduction

### Background

Voluntary medical male circumcision (VMMC) has been shown to reduce risk of male acquisition of HIV by as much as 60% [1-3]. Based on this evidence, in 2007 the World Health Organization (WHO) and the Joint United Nations Programme on HIV/AIDS (UNAIDS) stated that “male circumcision should now be recognized as an efficacious intervention for HIV prevention” and that male circumcision should be promoted as an additional strategy in the prevention of HIV in men [4]. Thirteen countries in eastern and southern Africa with high HIV prevalence and low rates of male circumcision were prioritized for VMMC scale-up [5]. Significant technical and financial resources were provided by various funding and technical agencies, and the governments of these countries began a process of conducting situation analyses, drafting policies and protocols, and rolling out VMMC [5]. As a result of these efforts, by the middle of 2014 over 5.8 million VMMCs had been performed, more than half of which occurred in 2013 [6].

### Early Resumption of Sexual Intercourse After VMMC

Expert guidelines for VMMC recommend a 6-week period of abstinence from penetrative sex after VMMC in order to avoid the spread of sexually transmitted infections, including HIV [7]. The risk of early resumption of sex exists without proper counseling, education, and follow-up. In the Rakai, Uganda trial, about 11% of HIV-positive and HIV-negative participants reported having intercourse before certified wound healing, defined in this study as “when there was an intact healthy scar with no residual exudate or scab formation, and all sutures had been completely absorbed” [8]. Married men were more likely to resume sexual intercourse before certified wound healing whether or not they were HIV positive (28%) or HIV negative (29%). This was despite intensive pre- and post-VMMC counseling within the trial, indicating strong sociocultural desire for quick resumption of marital sexual relations. In the same study, it was observed that more female HIV-negative partners of HIV-positive VMMC recipients enrolled in the trial acquired HIV when the couple resumed sex early (more than 5 days prior to certified wound healing) as opposed to those who did not [9]. In an observational study more approximating realistic clinical VMMC situations in Nyanza, Kenya, 30.7% of all participants resumed sexual intercourse before wound healing, usually in the first 3 to 4 weeks after VMMC [10]. Again, being married or cohabiting was the strongest predictor of having early sex: 65.7% of married participants resumed sex before healing despite counseling efforts by clinicians. Finally, a recent study from Zambia found that 24% of circumcised men resumed sex early, nearly half of whom (46%) did so in the first 3 weeks [11].

Cultural norms play an important role in expectations surrounding sexuality, and this has implications for health education messaging. Qualitative research from Nyanza, Kenya, indicates that the postoperative abstinence period is spontaneously cited by uncircumcised men as a barrier to obtaining VMMC; in particular, the 6-week period was considered too long to abstain [12]. Younger men were worried that their female partners would seek sex elsewhere, while older men worried that it would not be possible to sleep in the same bed as their wives and abstain. In the formative phase of the current project, we used qualitative methods to understand how newly circumcised men and their female partners feel about the 6-week abstinence period after VMMC. Our results confirm that it is not always easy for couples to navigate this period [13]. They live in close quarters so cannot avoid sleeping in the same bed, alcohol (and drug) use sometimes impairs their judgment, and there are expectations of sexual activity among married couples that men say they feel obliged to live up to.

### Pre- and Postoperative Counseling and Education

All of the studies above have reiterated the need for proper counseling and education about the efficacy of VMMC and the need to maintain or adopt proper risk avoidance behavior after the procedure, particularly during the healing period. In the Rakai, Uganda, trial the authors concluded that “the association between resumption of sexual intercourse before complete wound healing and increased risk of male-to-female HIV transmission makes it imperative that circumcised men and their female partners are clearly instructed to abstain from intercourse until the wound is healed” [9]. In a pooled analysis of the three original efficacy trials, Mehta et al (2009) found that men reporting early sex did not have increased risk for HIV after 3 or 6 months [14]. However, the authors acknowledged that the intensive counseling involved in the context of the three studies is not likely to be replicated in real life, indicating the importance of continued vigilance regarding actual counseling practices and resulting behaviors. Further, more intensive counseling of married men, due to their increased likelihood of resuming sex early, was recommended by the authors.

A recent study indicated the effectiveness of a 180-minute theory-based risk reduction group counseling session in reducing sexual risk behavior following VMMC, although the study did not report on early resumption of sex [15]. This is a promising intervention for informing VMMC programs. However, there is also recognition that the lack of human resources in the VMMC scale-up countries presents a barrier to such intense services, particularly if repeated messaging is to occur [16]. This is particularly the case when countries turn to independent partners to carry out mass, one-off VMMC campaigns outside of the normal constellation of services. Clearly, more innovative strategies for communicating with and effectively altering behavior in men and their partners are needed. Further, given evidence of greater risk for resumption of sexual activity in married/cohabiting men, strategies for nonpenetrative sexual activity should be developed and included in such education.

### mHealth As a Self-Care Strategy

Mobile health (mHealth)—the use of mobile phone technology to deliver health care—has emerged as an important and appreciated complement to health care education delivered through traditional channels. Such technology may include the use of text messaging, video messaging, voice calling, and Internet connectivity. The potential for mHealth interventions to partially compensate for interpersonal services in resource-poor areas is enormous [17]. Although mHealth has been shown to be effective in medication adherence, clinical management, and behavior modification [18], the use of mHealth has primarily been restricted to developed countries. To date, there are few studies of the use of voice messages to reduce risky sexual behaviors. In fact, a Cochrane Review from 2013 found only one RCT of a telephone-delivered intervention (use of postexposure prophylaxis for rape victims) for preventing HIV infection in HIV-negative persons [19]. Further, most mHealth interventions are not theory-based, leading to poor results [20].

### South Africa

South Africa holds the dubious title of being the country with the highest number of HIV positive individuals – over 6 million – and it accounts for 16% of all new HIV infections in the world [21]. Based on antenatal data, HIV prevalence in the general population is estimated at 17.3% in South Africa [22], with incidence estimated at 1.43%. Transmission of HIV in South Africa is almost exclusively through heterosexual sex, thus heightening the importance of VMMC as a form of prevention [23].

South Africa has committed to rolling out medical circumcision as one source of protection from HIV [24]. Circumcision is available as part of a comprehensive service at district hospitals, and, in theory, HIV testing, counseling, and HIV education are provided before the procedure. However, the South African health system is struggling to maintain high-quality counseling services around male circumcision due to human resource issues [5].

The Coloured community, which accounts for 48.8% of the Western Cape’s population [25], has a growing HIV prevalence rate—7.5% according to 2012 antenatal data [22]. The heightened HIV risk to this population group lies at least partly in high illicit drug and alcohol use, which is associated with risky sexual behavior [26].

Mobile phone ownership in South Africa is nearly universal—97% of households have a mobile phone, with greater concentrations of ownership in the urban centers. Particularly within the urban setting there is little difference by income level in terms of access to phones [27]. Given this near-universal ownership, mobile phone technology has been found to be acceptable and feasible for HIV- and AIDS-related prevention and services [28,29] and is now used in several health-related text-reminder projects in South Africa [30].

### Objectives

The objective of this study was to test the effectiveness of a customized relay of audio clips on safe sexual behavior in consenting, recently circumcised men.

## Methods

### Overview

The study is a randomized controlled trial (RCT) with two arms. The control arm consists of standard of care for pre- and postoperative counseling offered to VMMC recipients by the South African Department of Health VMMC services. In the intervention arm, VMMC recipients will receive voice messages for 6 weeks following the operation in addition to the standard of care. Data collection began January 21, 2015, and is ongoing.

### Study Setting and Participants

The research will be done in seven clinics in Cape Town, in the Western Cape Province of South Africa. The study sites were chosen in conjunction with the provincial health department. The communities in each of the seven catchment areas are almost exclusively Afrikaans-speaking Coloured. The term Coloured refers to an official South African race group used in research and census data that is predominantly mixed ancestry. The term originated in the apartheid era but remains an important descriptor and label for a distinct community. More than 48% of the people who live in the Western Cape are classified as Coloured, mostly still living in defined communities that are at least 90% Coloured. Inclusion criteria are men aged 18 years and older who present for VMMC at one of the study clinics and indicate at the precounseling session that they possess a mobile phone and consent to participate in the study. There are no exclusion criteria.

### Standard of Care (Control Group)

The standard of care offered by the provincial circumcision team consists of the counseling session during the HIV testing and counseling procedure and a brief postsurgery counseling session where men are advised on how to care for the wound and ordered to go to their local clinic at 2 days and 7 days following surgery. They are reminded not to engage in penetrative sex until the mandatory wound-healing period of 6 weeks has passed. No further contact is sought other than if there are health complications such as swelling or infection.

### Intervention Group

The intervention group gets the standard of care plus the intervention program which consists of 38 audio messages delivered over the 42 days following surgery. Once men are randomized into the intervention group, the project manager passes on the participants’ mobile phone numbers, personal identification numbers, and dates of enrollment to the mHealth platform operator (the South African company Health Information Systems Program [HISP]). The mobile system will automatically call participants twice a day for the first 2 days, once a day for the next 4 weeks, and on alternative days in the last 2 weeks. Using the last 4 digits of their mobile number as their password, participants can listen to the message and, using their keypad, replay it if they do not understand the message. It is not possible to respond to the message. The platform is programmed to redial unanswered or busy numbers up to 3 times. Therefore, calls can be received at different times of the day.

The content and phasing of the messages for the mHealth intervention were developed collaboratively with former patients through focus group interviews, cognitive interviews, and discussions with health promotion experts at the Provincial Medical Office (research to be published separately) using classic behavior change theories. The messages were then developed into short audio clips of 30 to 120 seconds each (in English and Afrikaans). Based on the formative research, messages delivered over the 42 days are divided into four periods:

- Days 1-2: An intense 2 days of self-care messages (2 per day). The theme of these messages revolves around coping with pain and recuperation.
- Days 3-14: Mainly self-care messages (1 per day). The theme is around strategies and practical tips on pain and wound management.
- Days 15-28: Coping and inspirational messages (1 per day). The theme is around coping with the wound inspiring and encouraging them to include their partners into the recovery period.
- Days 29-42: Inspirational messages (triweekly). The theme is around offering alternatives to penetrative sex and inspiring them to complete the period penile penetration-free.

### Outcomes

The primary outcome is occurrence of sexual intercourse (vaginal or anal) at any time in the 42 days after the procedure. Secondary outcomes include (1) adoption of nonpenetrative sexual behaviors in the first 42 days after the procedure, (2) self-reported sexually transmitted infection symptoms at baseline and 42 days after the procedure (as a marker of unprotected sex), (3) sexual risk behavior at baseline and 42 days after the procedure, and (4) sexual risk propensity at baseline (as a control for risk-taking personality) [9].

### Sample Size

A total sample size of 1188 (inflated by 10% for loss to follow up, n=594 in each arm) is needed to detect a 10% reduction of penile sexual events in the intervention group with 90% power with an alpha of .05. The control event rate is estimated at 60% based on previous studies in similar populations.

### Recruitment

Participants are recruited at the clinics where MMC services are offered in the greater Cape Town area. There are two roaming VMMC teams in the area offering services 4 days per week. The research assistants are divided between the teams and follow them depending on where they are on a set weekly schedule.

### Allocation

The randomization sequence will be generated by a biostatistician using a computer-generated table of random numbers. Assignment sequences will be placed in consecutively numbered opaque sealed envelopes ensuring allocation concealment. The study numbers are allocated consecutively as the patients come into the waiting area and written in the top corner of the envelope, which is handed to the participant.

### Blinding

This will be a single-blind RCT: data collectors, researchers, and analysts will all be blind to the patient’s allocation to study arm. The envelopes are numbered consecutively by the fieldworkers, and at the end of each recruitment day, the team sends a list of the participants’ study ID numbers, mobile numbers, and preferred language of messages to an office-based study administrator who has the group allocation master list. The study administrator then logs on to the HISP website and registers the mobile phone details of the intervention group, who start getting messages the next day. At no stage does the team in the field know the allocation of any study number, and at no stage does the study administrator come into contact with the participants or their personal information.

### Data Collection

Data is collected through self-administered paper and pen questionnaires offered in either English or Afrikaans. The consenting participants fill out the questionnaire while they wait for their HIV testing and preoperative counseling. They are asked to come back to the clinic for a follow-up visit after 6 weeks. HIV status will be collected (coded) from files if consent is given by the participant. Completed questionnaires are processed and captured at the office by the data capturer and stored in a locked room. The completed questionnaires are linked to the participants through their study numbers only and not the electronic health platform.

### Follow-Up Procedures

Participants return to the same clinic where they were recruited (or a nearby convenient public space) after 6 weeks for a follow-up visit consisting of a questionnaire. Based on many years of panel studies in the population, the team has developed the following mechanism for ensuring high retention: as part of the consent process, the participant will complete a contact form including his phone number and the names and phone numbers of two people close to him. He will also be given an appointment card that indicates the date of the 42-day follow-up visit. In the 6 weeks following surgery, participants are contacted with a brief phone call at 1, 3, and 5 weeks to remind them of their follow-up date and to reschedule if necessary. If a participant does not show up for the follow-up visit, contact is attempted up to 3 times in the following 2 weeks to reschedule. If the participant is reached but unable to reschedule, he is invited to complete a subsample of the follow-up questionnaire (10 questions) via the telephone. If he is not located, he is considered lost to follow-up.

### Analysis

#### Descriptive Analysis

Data will be analyzed using Stata 13 statistical software (StataCorp LP) according to the intention-to-treat principle. Descriptive statistics will be used to summarize the data. Continuous outcome variables will be tested for normality using descriptive statistics (eg, histograms, qq plots, and box plots). If normally distributed, continuous variables will be presented as means and standard deviations; if not, they will be reported as medians and interquartile ranges. Categorical data will be presented as proportions. An alpha of .05 will be considered statistically significant, and 95% confidence intervals will be reported where appropriate. Baseline prognostic variables and possible confounders will be compared between intervention and control groups using the appropriate univariate statistical methods. Clinical imbalances will be considered in further adjustment regression models.

#### Inferential Analysis

The primary outcome—occurrence of any homosexual or heterosexual penetrative intercourse at any time in the first 42 days after VMMC—will be compared between the intervention and control groups. The primary analysis will be to detect a difference in proportions using an unadjusted binomial test. Additionally, generalized linear regression models for the analysis of binary outcomes will be used to study the effects of the intervention as well as possible synergistic effects, taking potential confounders into account if necessary (eg, age, religion, marital status, education, employment, depression). The final covariate model will include all variables known to have a meaningful impact on bias in the estimate of the treatment effect. The covariate adjusted relative risk for treatment effect and 95% confidence interval will be reported. A covariate adjusted absolute risk difference for the effect of treatment and the 95% confidence interval will also be presented. In addition, we will report number needed to treat.

Secondary outcomes will be analyzed in the same manner as the primary outcome. A per-protocol compared to intention-to-treat sensitivity analysis will be performed on the primary outcome.

Missing primary outcomes will be assumed to be missing at random; however, if more than 15% of all primary outcomes that should be available for analysis are missing, a sensitivity analysis will be undertaken in addition to the primary missing at random analysis and results compared.

### Other Substudies

There is a substudy embedded in the follow-up survey at 42 days which looks at how the study population reacts to the mobile system. The purpose is to evaluate the level of learnability and feasibility for the user in real life. This includes responsiveness to messages. Another study of nonresponse will be conducted comparing answers to a smaller number of questions (on exposure and outcome) of those lost to follow-up with those who remained in the cohort.

### Ethics and Dissemination

#### Ethical Issues

The three fundamental principles of research ethics—respect, beneficence, and justice—will be upheld through the use of an approved informed consent form, a completely confidential enrollment procedure and documentation system, and thorough ethical training and certification of all research staff that come in contact with participants. HIV results will be linked to individuals through a coded system. No interviewer will have access to an individual’s HIV test results. The phone messages will only be accessible by the intended recipient, who will be given a password. Thus, there is no potential for accidental revealing of the patient’s VMMC or HIV status or participation in the study. The study was approved by the Health Research Ethics Committee of Stellenbosch University (ref N14/08/108) and is registered in the Pan-African Clinical Trial Registry [PACTR201506001182385].

#### Informed Consent

The informed consent will be administered either one-on-one with the participants or in a group format. The group format will be followed up by a one-on-one session before signing. This consent process will be administered by a trained study recruiter. The staff member will offer to read the informed consent word-for-word to the participant. If the participant declines the offer, the staff member will give the participant ample time to read the consent form. When the participant has read the consent form, the staff member will go over the essential elements contained within. If the participant declines to read, all aspects of the informed consent will be reviewed in a language understood by the participant (English or Afrikaans). As part of the consent processes, the staff member will ask a series of open-ended questions to assess the participant’s understanding of the consent. If they fail to get more than 80% of the answers right, they will be asked to read the informed consent form once again; the recruiter can then focus on the issues that they answered incorrectly and try to clarify the information. Once the above procedure has been completed successfully, the study recruiter will read out the signature page to each participant before they sign the form. A copy of the signed form will be given to the participant.

#### Quality Control

Standard operating procedures were developed and key staff were trained formally in clinical research standards. A clinical monitoring visit was conducted by an external consultant and adaptations were made thereafter. All fieldworkers received training on good clinical practices.

#### Dissemination

Two publications from the development of the intervention are planned, and at least three publications on the effectiveness of the intervention and the substudies described above are planned. Results from the study will be published within three years of study completion in Creative Commons and open-access journals. The data will be owned by Stellenbosch University and protected under South African law. A description of the project and the variables will be provided to Swedish National Data Service, where the data will be stored.

## Results

Enrollment was completed on June 29, 2016. Follow-up of enrolled participants is ongoing. Loss to follow-up is under 10%. No interim analyses have been conducted.

## Discussion

### Summary

This trial will test a novel intervention developed with a participative, theory-based approach. If it is found to be effective, the intervention should have application to other areas of health care, particularly where human resource shortages are chronic.

### Limitations

There could be some response bias due to social desirability, meaning that those who got the messages may be more likely to report they did not have sex than those who did not get the messages (because they have been reminded repeatedly, not necessarily because they did not have sex). Additionally, the participant could potentially have healed completely before the official 6-week postoperative period ended, which would mean that penetrative sex was theoretically clinically safe. A patient could also unintentionally report his allocation status to a nurse during a clinical follow-up visit, thus contaminating the blinding. Finally, there is a possibility that going through the male circumcision procedure may disinhibit men from practicing safe sex such as using condoms with casual partners. The original study protocol included a 6-month follow-up to study the effects of the intervention on such risk compensation. However, funding did not allow for this and this arm was removed. Future studies should include such aspects where possible.

## Abbreviations

HISP: Health Information Systems Program
RCT: randomized controlled trial
UNAIDS: Joint United Nations Programme on HIV/AIDS
VMMC: voluntary medical male circumcision
WHO: World Health Organization
